# Heparin-Binding EGF-Like Growth Factor Induces Heart Interstitial Fibrosis via an Akt/mTor/p70s6k Pathway

**DOI:** 10.1371/journal.pone.0044946

**Published:** 2012-09-12

**Authors:** Hong Lian, Yuanwu Ma, Juan Feng, Wei Dong, Qing Yang, Dan Lu, Lianfeng Zhang

**Affiliations:** 1 Key Laboratory of Human Disease Comparative Medicine, Ministry of Health, Institute of Laboratory Animal Science, Chinese Academy of Medical Sciences and Peking Union Medical College, Beijing, China; 2 Key Laboratory of Human Diseases Animal Model, State Administration of Traditional Chinese Medicine, Institute of Laboratory Animal Science, Chinese Academy of Medical Sciences and Peking Union Medical College, Beijing, China; Cardiovascular Research Institute Maastricht, Maastricht University, The Netherlands

## Abstract

Heparin-binding epidermal growth factor-like growth factor (HB-EGF) is essential for maintaining normal function of the adult heart and is known to play an important role in myocardial remodeling. In the present study, we observed that heart-specific HB-EGF transgenic (TG) mice had systolic dysfunction with decreased fractional shortening (FS%), increased end-systolic diameter (LVIDs) at 5 months of age, increased heart fibrosis, and increased mRNA expression of Col1α1 and Col3α1 at 1, 3, 5 and 7 months of age compared to nontransgenic (NTG) littermates. However, the left ventricular anterior wall thickness at end-systole (LVAWs) of the TG mice was not different than the NTG mice. Phosphorylation levels of Akt, mTor and p70s6k were increased due to HB-EGF expression in TG mice compared with the NTG mice at 3 and 7 months of age. Additionally, activated Akt, mTor and p70s6k were co-localized with vimentin to cardiac fibroblasts isolated from TG mice. Furthermore, HB-EGF significantly increased phosphorylation levels of Akt, mTor and p70s6k and increased expression of type I collagen in cultured primary cardiac fibroblasts. Rapamycin (Rapa) and CRM197, inhibitors of mTor and HB-EGF respectively, could inhibit the expression of type I collagen in the cultured primary cardiac fibroblasts and Rapa suppressed interstitial fibrosis of the heart tissues *in vivo*. In addition, a BrdU assay showed that HB-EGF increased proliferation of cardiac fibroblasts by 30% compared with cells without HB-EGF treatment. HB-EGF-induced proliferation was completely diminished in the presence of Rapa. These results suggest that HB-EGF induced heart fibrosis and proliferation of cardiac fibroblasts occurs through activation of the Akt/mTor/p70s6k pathway.

## Introduction

Heparin-binding epidermal growth factor-like growth factor (HB-EGF), a member of the EGF-family of growth factors, is a type I transmembrane protein composed of signal peptide, heparin-binding, EGF-like, juxtamembrane, transmembrane, and cytoplasmic domains [1], [2]. The membrane-bound proHB-EGF is cleaved at the juxtamembrane domain, causing shedding of mature HB-EGF. Full-length proHB-EGF is biologically active as a juxtacrine growth factor that signals neighboring cells in a non-diffusible manner [3]. Mature HB-EGF is a potent mitogen and stimulator for a number of cell types, including vascular smooth muscle cells, fibroblasts, and keratinocytes [4], [5].

HB-EGF is expressed in a variety of tissues, including the lung, heart, brain, and skeletal muscles [6], [7]. Moreover, HB-EGF is involved in a number of physiological and pathological processes, including heart development and maintenance [8], skin wound healing [9]–[11], eyelid formation [12], cardiac hypertrophy [13], smooth muscle cell hyperplasia [14], kidney collecting duct morphogenesis [15], blastocyst implantation [16], [17], pulmonary hypertension [18], and oncogenic transformation [19]. HB-EGF binds to and activates the EGF receptor (EGFR) via autophosphorylation, which recruits signaling molecules. However, HB-EGF activity may have various functions depending on the cellular context and activates different cell signal transduction networks.

In normal physiological conditions HB-EGF is found in the adult heart, and is further up-regulated under pathological conditions including cardiac hypertrophy or myocardial infarction. Although insulin-like growth factor (IGF), hepatocyte growth factor (HGF), and HB-EGF are cardiogenic factors and the therapeutic effects of IGF and HGF have been well demonstrated [20], [21], it is uncertain whether up-regulation of HB-EGF following cardiac injury is a protective or pathological effect during heart remodeling. To clarify this issue, we constructed a transgenic (TG) mouse with heart-specific overexpression of HB-EGF. Hearts of TG mice showed systolic dysfunction with increased collagen deposition and interstitial fibrosis. A number of previous studies have shown that HB-EGF is related to fibrosis [22]–[27], however, little is known regarding the molecular mechanisms and the responsible cell-type (cardiomyocytes or cardiac fibroblasts) for HB-EGF-induced fibrosis. Initially, all studies regarding signal transduction of heart fibrosis were performed in whole hearts. In the present study, we investigated the signaling pathways involved in HB-EGF-induced fibrosis, focusing on cardiac fibroblasts of HB-EGF TG mice and primary culture fibroblasts.

## Results

### Generation of TG Mice with Heart-specific Overexpression of HB-EGF

**Figure 1:**
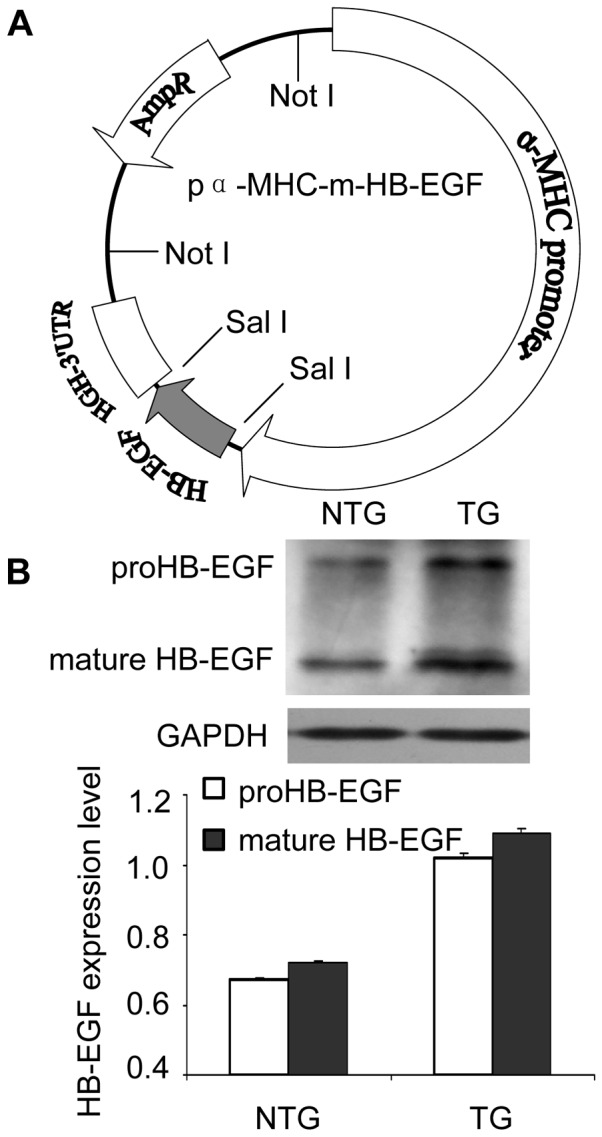
Generation of TG mice. The HB-EGF TG construct was generated by the insertion of a target gene under the control of the α-MHC cardiac-specific promoter (A), and the TG mice were created using a microinjection method. Mouse lines with overexpression of HB-EGF (proHB-EGF and mature HB-EGF) were selected using a western immunoblot procedure and normalized with GAPDH (B).

The transgenic plasmid (Figure 1 A) composed of the α-MHC promoter and mouse HB-EGF cDNA was linearized using Not I digestion. TG mice were generated by microinjection and the expression of HB-EGF was screened using western immunoblot (Figure 1 B). Both of the proHB-EGF and mature HB-EGF were increased to 1.5 fold in the heart tissues of TG mice compared with that of the nontransgenic (NTG) mice.

### Systolic Dysfunction and Interstitial Fibrosis in the Hearts of HB-EGF TG Mice

**Figure 2:**
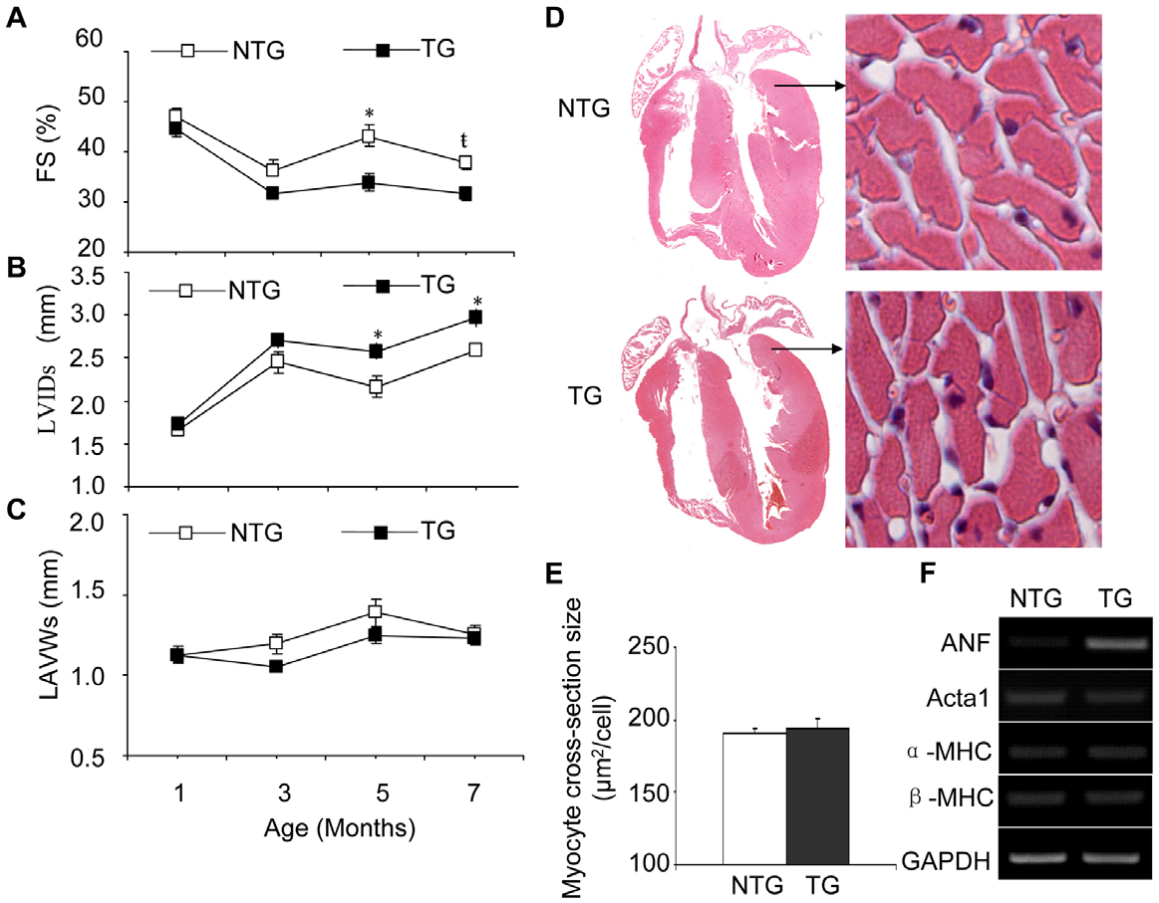
Echocardiography and H&E staining. TG mice and their NTG littermates were analyzed using M-mode echocardiographic analyses at 1, 3, 5, and 7 months of age. Fractional shortening (FS%) was decreased by the expression of HB-EGF compared with that of NTG mice (A). The left ventricular end-systolic diameter (LVIDs) was increased by the transgenic expression of HB-EGF compared with that of NTG mice (B), while left ventricular anterior wall thickness at end-systole (LVAWs) was not different (C). The whole heart (original magnification ×4) from TG and NTG littermates were sampled and stained using H&E and observed with a microscope. Cardiomyocytes (original magnification ×400) from the same areas of the left ventricle from TG and NTG littermates were compared to detect changes of myocyte cross-section size (D). About 50 cells per sample slice were randomly selected to calculate the average area of a single cell size (E, *n* = 4). *P<0.05 *vs.* NTG mice; 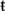 *P*<0.001 *vs.* NTG mice.

**Figure 3:**
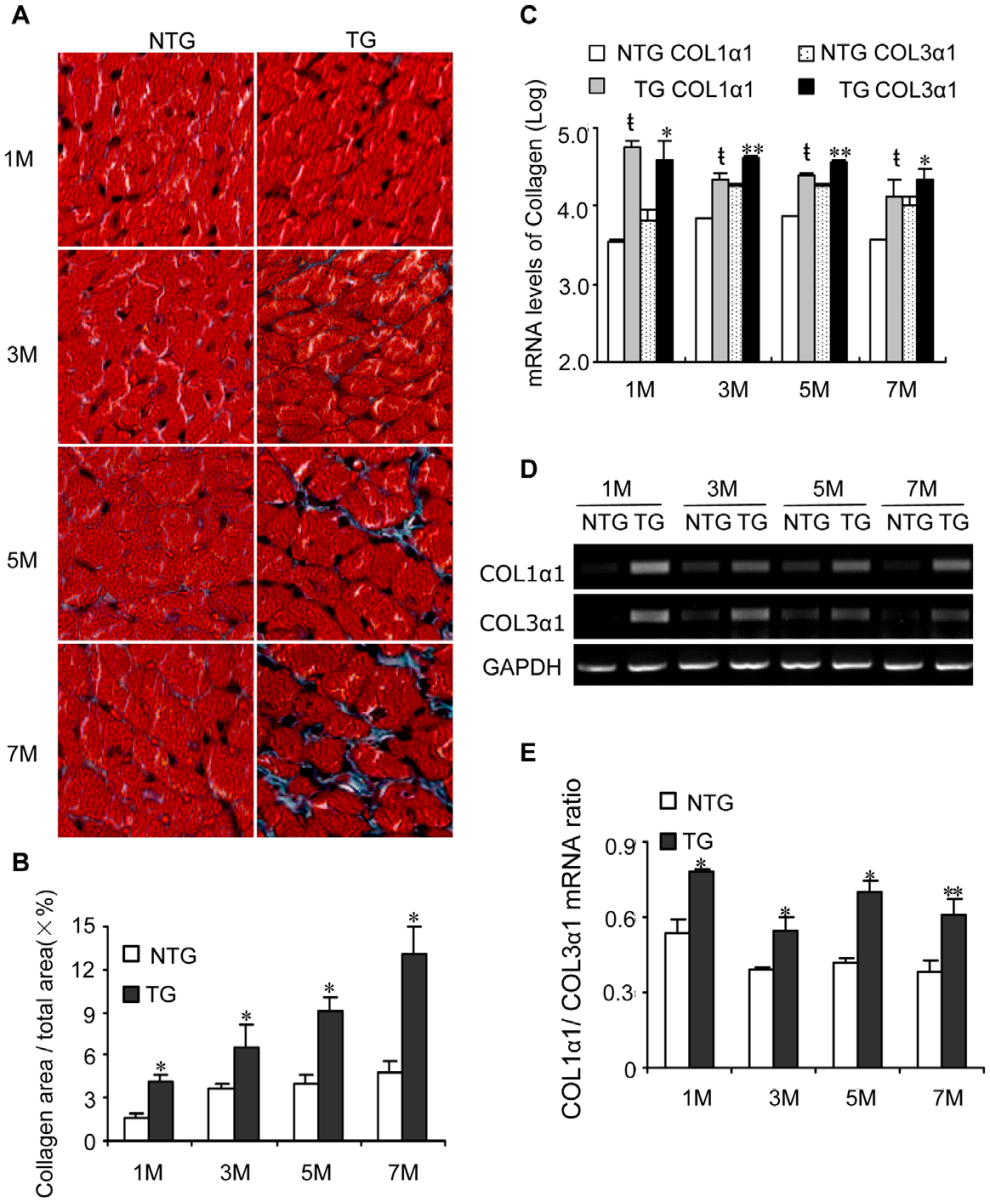
Determination of cardiac fibrosis. Mice were sacrificed at 1, 3, 5 and 7 months of age, and heart tissues were examined for pathological changes and collagen expression. Heart fibrosis, as determined by Masson staining (A, original magnification ×200). The fibrosis area of the section was quantified using image-Pro Plus 6.0 software, the ratio of collagen area and total area (×%) was counted and compared (B). *P<0.05 *vs.* NTG mice. The synthesis of Col1α1 and Col3α1 was detected using quantitative real-time PCR (C) and RT-PCR (D). 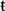 *P*<0.001 *vs.* Col1α1 of NTG mice; *P<0.05 *vs.* Col3α1 of NTG mice; **P<0.001 *vs.* Col3α1 of NTG mice. The Col1α1/Col3α1 ratio was compared between NTG and TG mice (E). *P<0.05 *vs.* NTG mice; **P<0.001 *vs.* NTG mice.

Ventricular size and function of the TG and NTG mice were assessed using echocardiography. The changed parameters of M-mode echocardiography from the TG mice and the NTG littermates at 1, 3, 5, and 7 months of age are summarized in Figure 2 and all the echocardiographic parameters, heart rate and the ratio of heart weight to body weight for the mice at 7 months of age were summarized in Table 1. Alterations in ventricular size and functional parameters were observed from 5 months of age in the TG mice compared with NTG mice. FS% was decreased by 21% (*n* = 12, *P*<0.05) and the LVIDs was increased by 19% (*n* = 12, *P*<0.05) in TG mice at 5 months of age compared with NTG littermates (Figure 2 A, B). The LVAWs in TG mice was not different than NTG littermates at all time points (Figure 2 C). Cardiomycyte size from TG heart tissues was not different when comparing hematoxylin and eosin (H&E) staining (Figure 2 D, E, *n* = 4). The expression level of ANF was increased obviously, other than the expression levels of Acta1, α-MHC and β-MHC in the heart of TG mice compared with that of the NTG mice (Figure 2 F, *n* = 3). Masson staining showed that interstitial fibrosis occurred in hearts excised from TG mice (Figure 3 A, B, *n* = 4). Quantitative real-time PCR and the RT-PCR results also indicated that HB-EGF TG mice had increased mRNA expression of Col1α1 and Col3α1 compared with NTG littermates (Figure 3 C, D), and also the Col1α1/Col3α1 ratio was elevated (Figure 3 E).

**Table 1:** Echocardiographic characteristics of NTG and TG mice at 7 months of age.

Parameters	NTG (*n* = 12, 7 m)	TG (*n* = 12, 7 m)
LVIDd, mm	4.17±0.07	4.33±0.09
LVIDs, mm	2.59±0.05	2.97±0.09*
LVVd, µL	77.50±2.88	85.41±4.04
LVVs, µL	24.61±1.16	34.78±2.75*
LVPWd, mm	0.72±0.03	0.74±0.04
LVPWs, mm	1.15±0.04	1.09±0.04
LVAWd, mm	0.81±0.02	0.86±0.03
LVAWs, mm	1.26±0.05	1.23±0.04
EF%	68.19±1.07	60.49±1.97**
FS%	37.80±0.87	31.64±1.32**
HR	455.08±20.99	463.75±12.16
HW/BW(mg/g)	5.42±0.17	5.52±0.41

LV: left ventricular; LVIDd: LV end-diastole diameter; LVIDs: LV end-systole diameter; LVVd: LV end-diastolic volume; LVVs: LV end-systole volume; LVPWd: LV posterior wall at end-diastole; LVPWs: LV posterior wall at end-systole; LVAWd: LV anterior wall at end-diastole; LVAWs: LV anterior wall at end-systole; EF%: percent ejection fraction; FS%: percent fractional shortening; HR: heart rate; HW/BW: heart weight/body weight.

* *P*<0.05 *versus* NTG mice;

** *P*<0.001 *versus* NTG mice.

### TG expression of HB-EGF Increased Phosphorylation of Akt, mTor and p70s6k in Cardiac Fibroblasts

**Figure 4:**
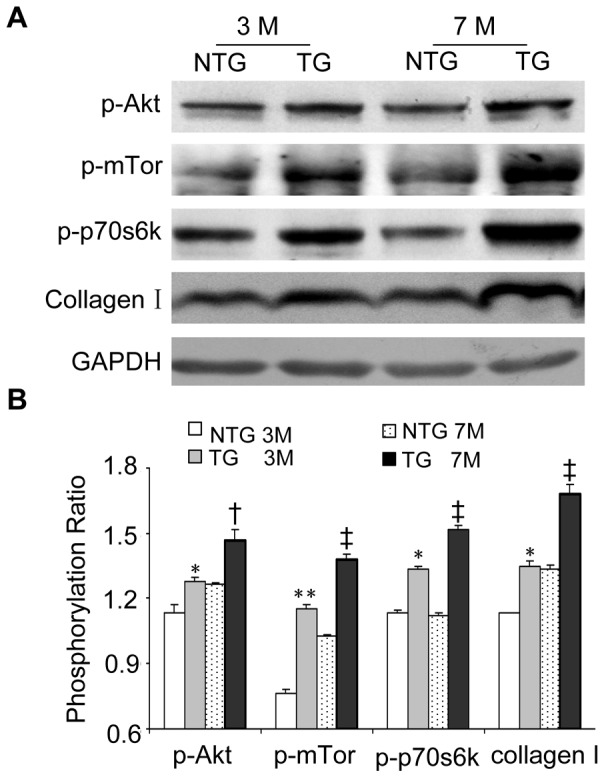
Determination of the phosphorylation levels of Akt, mTor and p70s6k in heart tissues. Mice were sacrificed at 3 and 7 months of age, and heart-tissue samples were homogenized in RIPA buffer. Phosphorylation levels of Akt, mTor and p70s6k were detected using western immunoblot (A). The relative intensities were detected using Image J and compared with those of NTG mice (B). *P<0.05 *vs.* NTG mice at 3month; **P<0.001 *vs.* NTG mice at 3month; †*P*<0.05 *vs.* NTG mice at 7month; ‡*P*<0.001 *vs.* NTG mice at 7month.

**Figure 5:**
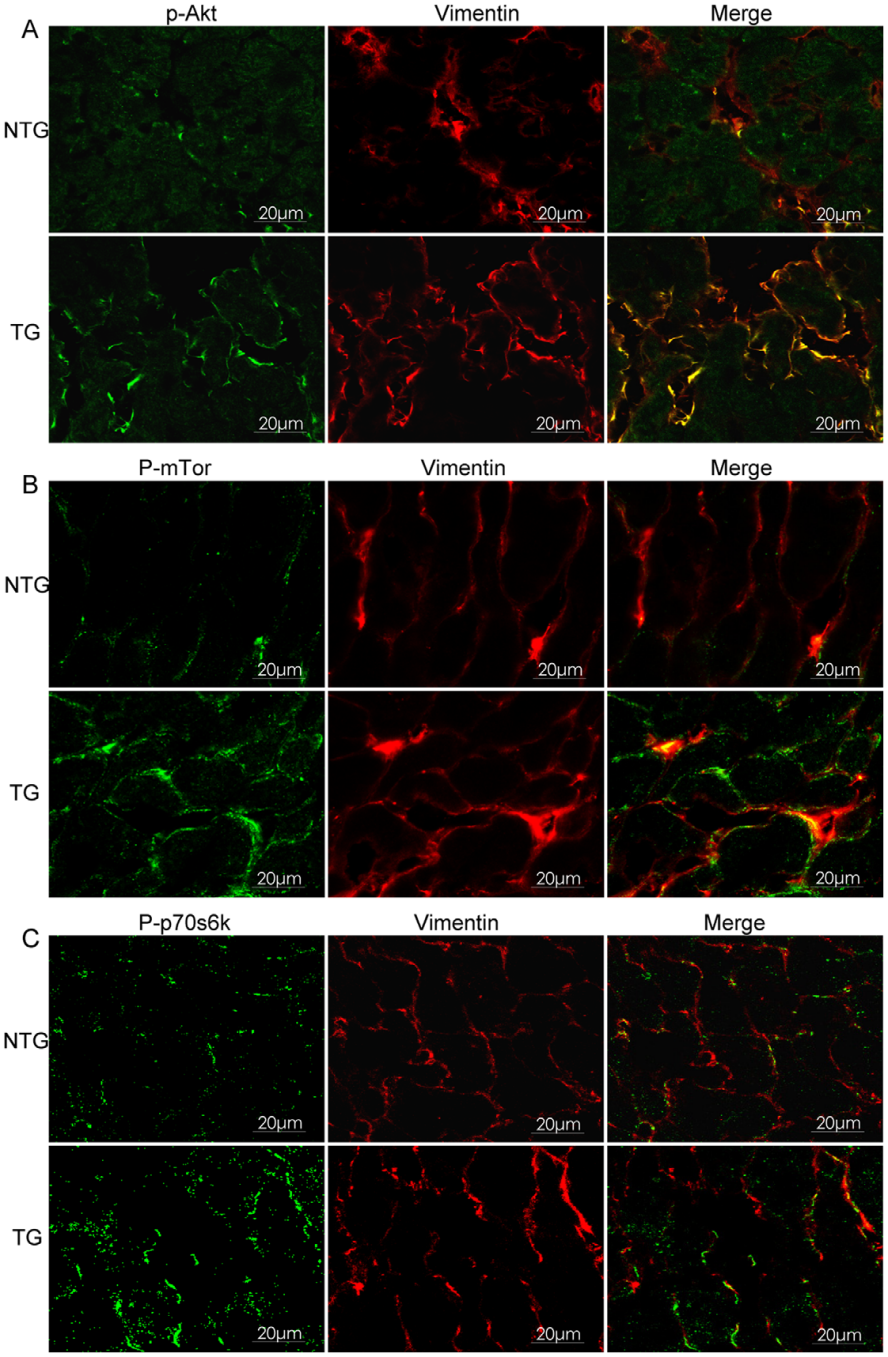
Localization of activated Akt, mTor and p70s6k in mouse heart tissues using double immunofluorescence confocal staining at 7 months of age. Cell localization of p-Akt (A), p-mTor (B) and p-p70s6k (C) were observed using confocal laser- scanning microscopy (Leica TCS SP2, Germany). Scale bar = 20 µm.

Western immunoblot of total heart protein at 3 and 7 months of age indicated that phosphorylation of Akt, mTor, p70s6k and the expression of collagen I were increased in the TG mice compared with NTG littermates (Figure 4 A, B). Additionally, we compared double immunofluorescence staining of sections from the mice hearts with anti-vimentin, which is a marker of cardiac fibroblasts, and phosphorylated Akt (Ser473), mTor (Ser2448) or p70s6k (Thr389) antibodies. These results showed that activated Akt, mTor and p70s6k were predominantly co-localized with the marker for cardiac fibroblasts, increased phosphorylation levels of Akt, mTor and p70s6k was evident in TG mice (Figure 5 A–C), suggesting that HB-EGF induced the activation of Akt, mTor and p70s6k predominantly in cardiac fibroblasts.

### mTor is Essential for HB-EGF Induced Cardiac Fibroblast Collagen Synthesis and Proliferation

**Figure 6:**
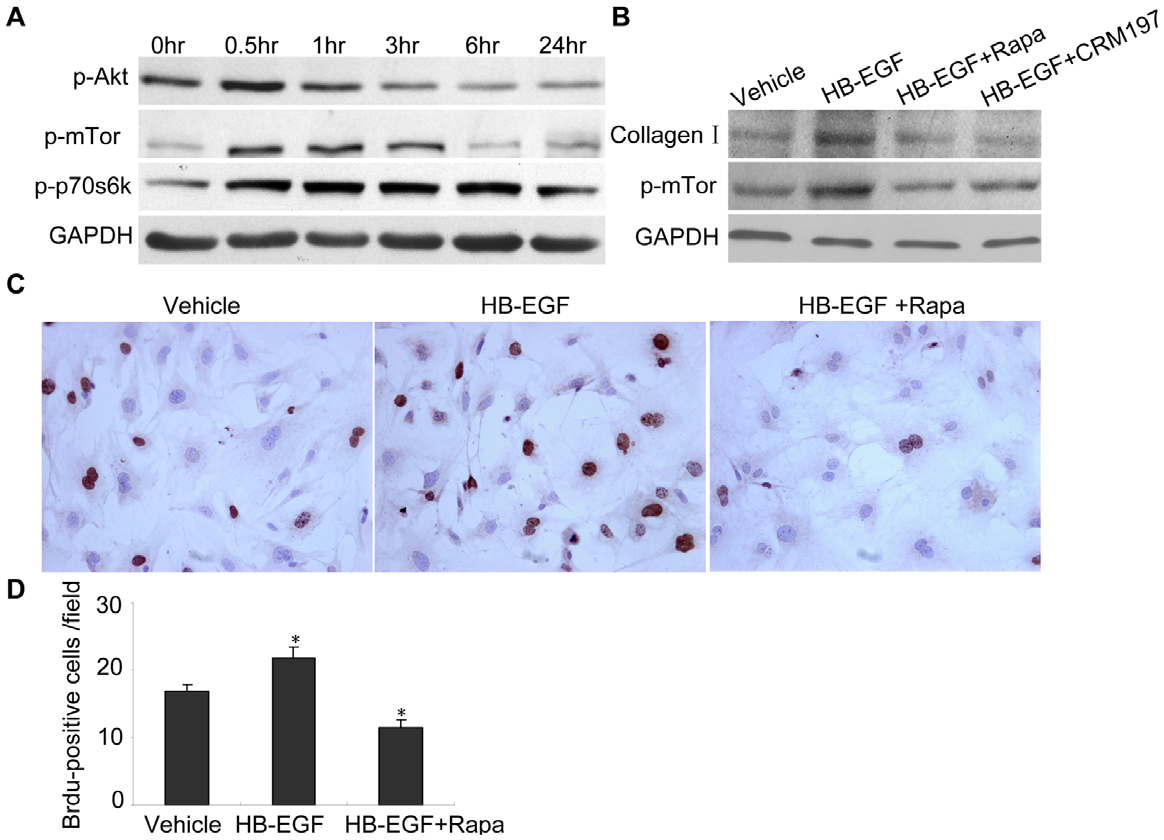
HB-EGF induced proliferation of cultured primary cardiac fibroblasts and synthesis of collagen *in vitro*. Cultured primary cardiac fibroblasts were treated with HB-EGF at various time points. Phosphorylation of Akt, mTor and p70s6k was analyzed using western immunoblot (A). The cultured primary cardiac fibroblasts were treated with DMSO (vehicle control), HB-EGF, HB-EGF + Rapa, and HB-EGF +CRM197. Expression of collagen I was then detected after treatment for 72 hr and phosphorylation of mTor was detected after treatment for 2 hr using western immunoblot (B). The cell proliferation was detected using BrdU staining and the BrdU-positive cells were counted from six sections (C, D). *P<0.05 *vs.* NTG mice; **P<0.001 *vs.* NTG mice.

**Figure 7:**
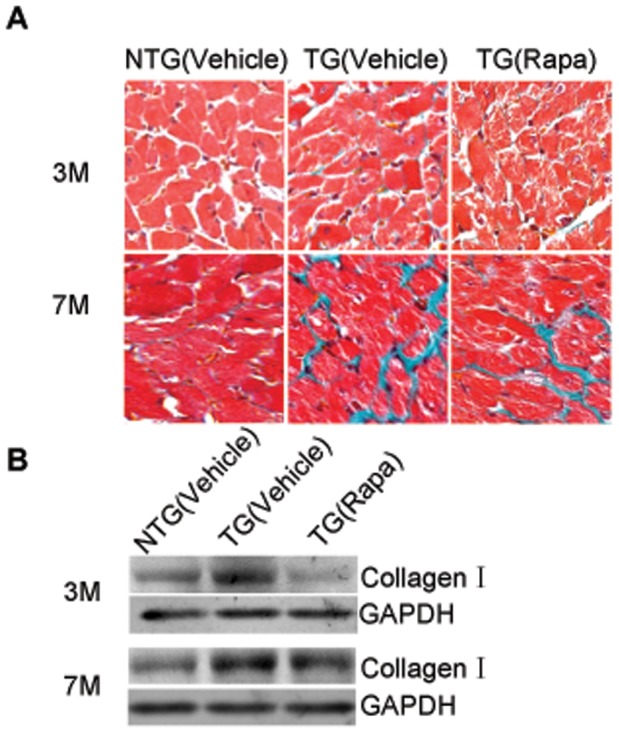
Rapa inhibited HB-EGF induced heart interstitial fibrosis and synthesis of collagen I in vivo. The mice were treated with vehicle or 5 mg/kg Rapa every second day by intraperitoneal injection for five times. Masson staining (A, *n* = 3) and western immunoblot (B, *n* = 3) were detected. Original magnification ×200.

Phosphorylation of Akt, mTor and p70s6k was stimulated by HB-EGF treatment in primary cultured cardiac fibroblasts. Phosphorylation of Akt was activated at 30 min, and the phosphorylation of mTor was strongly apparent for 3 hr, while the phosphorylation of the downstream factor p70s6k was apparent for 24 hr in the presence of HB-EGF (Figure 6 A). HB-EGF induced synthesis of collagen I and enhanced phosphorylation of mTor in primary culture cardiac fibroblasts, which was inhibited by Rapa or CRM197 (Figure 6 B). HB-EGF also increased the proliferation of primary cultured cardiac fibroblasts by 30% compared to cells without HB-EGF treatment, and HB-EGF–induced proliferation was completely diminished in the presence of Rapa (Figure 6 C, D). Furthermore, heart interstitial fibrosis induced by HB-EGF was also inhibited in the TG mice which were treated with Rapa (Figure 7 A, B). These results suggest that mTor is essential for HB-EGF induced cardiac fibroblast collagen synthesis and proliferation.

## Discussion

Myocardial remodeling, including cardiomyocyte remodeling and extracellular matrix deposition, is an important process contributing to morphological and functional adaptation during the acute phases of cardiac injury. It is well known that HB-EGF is related to myocardial remodeling, however, previous studies regarding HB-EGF in myocardial remodeling were more focused on functional cardiomyocyte studies. Ushikoshi *et al.* first proposed that HB-EGF exacerbates remodeling following myocardial infarction by the activation of non-cardiomyocytes (mainly cardiac fibroblasts) [22]. Furthermore, two previous reports confirmed that the EGFR, a receptor for HB-EGF, was observed mostly in the interstitial area, primarily in fibroblasts [23], [24]. Additionally, Blaine *et al.* demonstrated that EGFR signaling regulates fibrogenesis in TG mice with pancreatic-specific expression of HB-EGF [28]. These results suggest that HB-EGF may be involved in myocardial remodeling through the regulation of fibroblasts. Moreover, HB-EGF mRNA was up-regulated in the peri-infarct region of the remnant kidney model, suggesting a regulatory role in myofibroblast transformation [25].

Elevated HB-EGF expression results in stromal expansion progression to severe fibrosis of the endocrine and exocrine pancreas [26]. Moreover, HB-EGF is co-expressed with ADAM17 in renal interstitial fibrosis in a rat model of renal ischemia–reperfusion injury [27]. Live-specific expression of HB-EGF accelerated the proliferation of hepatocytes following partial hepatectomy [29]. Our previous work indicated that HB-EGF expression was increased with the development of dilated cardiomyopathy (DCM) in a cTnT^r141w^ transgenic model, and treatment with ginsenoside Rb1 attenuated HB-EGF expression and heart fibrosis [30]. These results suggest that HB-EGF is related to fibrosis in various tissues.

In the current study, HB-EGF TG mice had decreased FS% and increased LVIDs, while the LVAWs of TG mice showed no obvious differences compared with NTG littermates (Figure 2 A–C). H&E staining suggested that the cardiomyocyte size of TG heart tissues was not different than NTG mice (Figure 2 D, E). The increased expression of fetal gene of ANF might be a compensatory response to dysfunction of the heart of the TG mice. Heart tissues of the TG mice had significantly increased expression of Col1α1 and Col3α1 mRNA after one month, as well as at three, five and seven months of age, due to TG overexpression of HB-EGF (Figure 3 C, D). Additionally, the Col1α1/Col3α1 ratio was elevated in the heart of TG mice than that of NTG, which suggested that the myocardium of TG mice turn into stiff [31], [32]. Duration increased the expression of Col1α1 and Col3α, resulting in the deposition of collagen and leading to interstitial fibrosis (Figure 3 A, B). These results suggest that HB-EGF is involved in the development of interstitial fibrosis rather than hypertrophy of the heart. Additionally, mast cells were detected in the heart of NTG and TG mice and the quantitation of the mast cell was listed (Figure S1). In the heart of TG mice has no obviously mast cell degranulation prolife from either the number of mast cell or the edge of the mast cell. We detected the expression levels of the mRNA for ADAM9, 10, 12 and 17 [33] in the heart tissues from NTG and TG mice by RT-PCR, which were implicated as shedding enzymes of proHB-EGF. The result indicated that the transgenic expression of HB-EGF did not increase ADAMs expression in heart tissues obviously (Figure S2).

Previous reports have shown that HB-EGF induced cell proliferation in cerebral cortical cultures through the MEK/Erk and Akt pathways [34] and induced proliferation of vascular smooth muscle cells via Erk1/2, Akt, and p70s6k [35]. Herein, we determined phosphorylation levels of Erk1/2 and Akt in the heart, and the results showed that TG overexpression of HB-EGF did not regulate Erk1/2 phosphorylation (data not shown), however phosphorylation of Akt (Ser473) was increased by TG overexpression of HB-EGF compared with NTG hearts, while Akt (Ser308) was not changed (Figure S3). Furthermore, the phosphorylation of mTor and p70s6k, the downstream elements of Akt, were also increased significantly at 3 and 7 months (Figure 4). Phosphorylated Akt, mTor and p70s6k was predominantly localized in cardiac fibroblasts, which were labeled using anti-vimentin (Figure 5 A–C). Conserved Ser/Thr kinase mTor is a multifunctional protein involved in the regulation of cell growth, proliferation, and differentiation [36]–[38]. Previous studies have shown that Rapa inhibits hepatic stellate cell proliferation and attenuates hepatic fibrosis [39]–[43]. The mTor/p70s6k pathway plays an important role in regulating collagen I synthesis and extracellular matrix deposition in fibroblasts and hepatic stellate cells [44], [45]. Inhibition of mTor with Rapa reduced the number of interstitial fibroblasts and myofibroblasts in an obstructive nephropathy rodent model [46]. Our results indicated that HB-EGF induced phosphorylation of Akt, mTor and p70s6k and increased the proliferation and synthesis of collagen I synthesis of primary culture cardiac fibroblasts. Additionally, HB-EGF-induced proliferation and collagen I synthesis in the primary cultures and transgenic heart tissues were diminished by the mTor inhibitor, Rapa (Figure 6 and Figure 7). These results suggest that mTor is essential for HB-EGF-induced cardiac fibroblast collagen synthesis and proliferation, and the Akt/mTor/p70s6k pathway plays an important role in heart fibrosis.

Development of fibrosis has been shown to be a major component in several diseases including the heart, kidney, liver, lung and pancreas. However its role in the progression of these disorders remains unknown. The findings presented herein may be useful for the development of new therapeutics as well as for elucidating the mechanisms of HB-EGF-induced fibrosis during cardiac disease.

## Materials and Methods

### Animals

The Institutional Animal Care and Use Committee of the Chinese Institute of Laboratory Animal Science (GC05-009) approved all animal procedures, and the mice were housed in an Association for Assessment and Accreditation of Laboratory Animal Care (AAALAC) accredited facility. The TG founder mice were mated with wild type C57BL/6J mice to produce TG generations (12 generations), which were used for phenotypic analyses.

### Plasmid Construction and Preparation of TG Mice

Full-length mouse HB-EGF cDNA was cloned from C57BL/6J mouse heart tissue using reverse transcription–polymerase chain reaction (RT-PCR) and confirmed by sequencing analyses. Mouse HB-EGF cDNA was inserted downstream of the α-MHC promoter (Genbank accession no. U71441, clone 26). Linearized transgenic DNA fragments were gel purified, dissolved in Tris–HCl–EDTA at a final concentration of 5 ng/µl, and injected into the pronucleus of fertilized zygotes harvested from C57BL/6J mice using conventional methods used for the generation of TG mice [47]. Genotypes of the TG mice were determined using PCR with 35 cycles of denaturation at 94°C for 30 sec, annealing at 58°C for 30 sec and extension at 72°C for 30 sec to amplify the desired 491 base pair fragment of the HB-EGF transgene. The following HB-EGF primers were used: 5′-GTCGGTGATGCTGAAGCTC and 5′-CTACAGCCACCACAGCCAAG.

### Echocardiography

Mice were anesthetized with tribromoethanol (0.18 ml/10 g of body weight, i.p.) and echocardiographic images were obtained with a VisualSonics Vevo 770 system (Canada). M-mode echocardiography of the left ventricle was recorded at the tip of the mitral valve apparatus using a 30-MHz transducer (Vevo770, Canada) [48]. The experimental mice in each group (same number of male and female) were 1, 3, 5, or 7 months old.

### Histology and Immunofluorescence Observations

HB-EGF TG mice and NTG littermates were sacrificed at 1, 3, 5, and 7 months of age, and hearts were excised. Myocardial tissues (2 mm thickness) were fixed in formalin and embedded in paraffin. Lengthwise sections (4 µm thickness) were stained with H&E for microscope observation and measurement of myocyte cross-section size using Aperio ImageScope v8.2.5 software. The sections were stained with Masson staining and quantified using image-Pro Plus 6.0 software. Frozen sections of heart tissues from mice at 7 month of age were prepared in 10 µm thick sections and fixed in ice-cold acetone for 15 mins, blocked, and incubated with antibodies to phospho-Akt (Ser473)/phosphor-mTor (Ser2448)/phosphor-p70s6k (Thr389)(1∶50, Cell Signaling Technology), and vimentin (1∶100, Abcam) overnight at 4°C, respectively. Following washing with phosphate buffered saline (PBS), sections were incubated with secondary Alexa 488-conjugated goat anti-rabbit IgG (1∶100, Invitrogen) and DyLight-conjugated, affinity-purified anti-mouse IgG (1∶100, KPL) for 30 mins at 37°C. The sections were then rinsed again with PBS and mounted in ProLong Gold anti-fade reagent (Invitrogen). The sections were observed and analyzed using confocal laser-scanning microscopy (Leica TCS SP2, Germany).

### RT-PCR and Quantitative Real-time PCR Analyses

Total RNA was isolated from 10 mg of tissue from mice hearts using Trizol Reagent (Invitrogen). First-strand cDNA was synthesized according to the Superscript III reverse transcriptase manufacturer’s protocol (Invitrogen). Expression levels of mRNA for Col1α1, Col3α1, ANF, Acta1, α-MHC and β-MHC were determined using RT-PCR, and GAPDH was used for normalization. Primers used included Col1α1, 5′-CCTGCCTGCTTCGTGTAAACT and 5′-TTGGGTTGTTCGTCTGTTTCC; Col3α1, 5′-GGCAGTGATGGGCAACCT and 5′-TCCCTTCGCACCGTTCTT; ANF, 5′- ATGGGCTCCTTCTCCATCAC and 5′- TTATCTTCGGTACCGGAAGCTG; Acta1, 5′- CCGACCCCGTCACCAGGGTG and 5′- ATCCAACACGATGCCGGTG; α-MHC, 5′- TGCTGAGGGAACAGTATGAA and 5′- TCTGTATGGCATCCGTCTC; β-MHC, 5′- ACAGAGGAAGACAGGAAGAACC and 5′- GCTTGTTGACCTGGGACTC; and GAPDH, 5′-CAAGGTCATCCATGACAACTTTG and 5′-GTCCACCACCCTGTTGCTGTAG.

For real-time PCR, total RNA normalized by the PCR of GAPDH was assayed using one step RT-PCR in a 20 µl reaction mixture (QIAGEN QuantiTect SYBR Green RT-PCR kit). Primers were the same as used for semi-quantitative RT-PCR and the conditions consisted of a denaturation step at 95°C for 20 s and 40 cycles of thermal cycling of 95°C for 30 s, 59°C for 30 s and 72°C for 30 s. Incorporation of SYBR fluorescent dye into DNA double-strands was monitored and analyzed using a Roche LightCycler3.5 system. The amplification fragments of Col1α1 and Col3α1were used as real-time PCR standards by adjusting to a concentration gradient of 1×10^8^ copies/µl, 1×10^7^ copies/µl, 1×10^6^ copies/µl, 1×10^5^ copies/µl, 1×10^4^ copies/µl, 1×10^3^ copies/µl, 1×10^2^ copies/µl and 1×10^1^ copies/µl; the DNA fragment with known copies was used as a standard to calculate the copy number of the heart mRNA of the NTG and the TG mice.

### Western Immunoblot

Cardiac tissues from mice were lysed in RIPA buffer (50 mM Tris, pH 7.4, 150 mM NaCl, 1% Triton X-100, 1% sodium deoxycholate, 0.1% SDS, 1 mM EDTA, and protease inhibitor cocktail) using homogenization at 20,000 rpm for 1 min and centrifugation at 12, 000 rpm at 4°C for 30 mins. Supernatants from the heart tissues or primary culture fibroblasts were incubated with loading buffer in boiling water for 5 mins, separated on a SDS-PAGE gel, transferred onto a nitrocellulose membrane and blocked for 1 hr with 5% skim milk or BSA. The membrane was incubated overnight with antibodies specific to HB-EGF (Santa), phospho-Akt (Ser473), phospho-Akt (Ser308), phospho-mTor (Ser2448), phospho-p70s6k (Thr389) (Cell Signaling Technology), vimentin, or collagen type I (collagen I) (Abcam), followed by incubation for 1 hr with a 1∶20,000 dilution of horseradish peroxidase-conjugated anti-mouse or anti-rabbit IgG. The signal was visualized by enhanced chemiluminescence (Santa Cruz) and analyzed using Gel-Pro Analyzer software. GAPDH was used for internal protein normalization.

### Culture and Treatment of Primary Mouse Cardiac Fibroblasts

Primary mouse cardiac fibroblasts were isolated from 1∼3 day old mice. Briefly, hearts were placed in a Petri dish with cold PBS. The hearts were minced to approximately 1 mm^3^ or even small pieces. These pieces were digested at 37°C in collagenase II (0.05%) about 10 min and centrifuged at 2,000 rpm for 5 min. The pellet was dispersed in 100-mm culture dish with DMEM medium supplemented with 10% fetal bovine serum (FBS), 100 units/ml of penicillin, and 100 µg/ml of streptomycin. The primary mouse cardiac fibroblasts from the second to the fourth passage were used in this experiment. The cardiac fibroblasts were treated with 10 ng/ml recombinant human HB-EGF (HB-EGF, Sigma, E4643) for 0, 0.5, 1, 3, 6, or 24 hr to detect phospho-Akt, phospho-mTor and phospho-p70s6k. The cultured primary cardiac fibroblasts were treated with DMSO (vehicle control), HB-EGF, HB-EGF + Rapa (10 nM), and HB-EGF +CRM197 (200 ng/ml) [49] respectively. Expression of collagen I was then detected after treatment for 72 hr and phosphorylation of mTor was detected after treatment for 2 hr using western immunoblot. The cells were then harvested, washed with PBS in duplicate, and lysed with RIPA buffer for analyses.

### The Mice Treated with Rapamicin

Rapa (ALEXIS BIOCHEMICALS) was dissolved in DMSO (25 mg/ml). The mice at 3 and 7 months of age were treated with vehicle (21.5%DMSO, 21.5% EtOH, and 57% sterile saline) or 5 mg/kg Rapa (21.5%DMSO/Rapa, 21.5% EtOH, and 57% sterile saline) every second day by intraperitoneal injection for five times. Body weights were recorded every time, the dosing was adjusted accordingly [50].

### BrdU Immunocytochemistry Staining

For primary mouse cardiac fibroblasts, cells were seeded onto 24-well plates containing round cover slides. Following treatment with HB-EGF (10 ng/ml) and/or Rapa (10 nM) for 24 hr, the cell-culture medium was replaced with 1% BrdU-labeling solution and incubated for 1 hr at 37°C. The cells were then stained according to the BrdU immunocytochemistry staining kit (Invitrogen) instructions.

### Statistical Analyses

Data were analyzed using unpaired two-tailed Student’s t-tests for two groups or one-way ANOVA for multiple groups followed by a Tukey’s post hoc analyses. Data are expressed as mean ± SEM from individual experiments. Differences were considered statistically significant at *P*<0.05.

## Supporting Information

Figure S1 **The phosphorylation levels of Akt (Ser308).** Mice were sacrificed at 3 and 7 months of age, and heart-tissue samples were homogenized in RIPA buffer. Phosphorylation level of Akt (Ser308) was detected using western immunoblot.
(TIF)

Figure S2 **The mRNA expression levels of ADAMs in the heart of NTG and TG mice.** Expression levels of mRNA for ADAM9, ADAM 10, ADAM 12 and ADAM 17were determined using RT-PCR, and GAPDH was used for normalization. Primers used included ADAM9, 5′- TGCCTCTCTGCGACTAAGGT and 5′- ACTCGGATGCTCCTCCTCAT; ADAM 10, 5′- AAACACCAGCGTGCCAAA and 5′- TTCAGCCAGAGTTGTGCGT; ADAM 12, 5′- GGGACCAGAGAGGAACTTACGA and 5′- CTTCTTGCCCGCATTTGA; ADAM 17, 5′- GTGGTTGGTGAGCCTGACTCTA and 5′- AAGCATCCTTCTCTTCGTTTGG; and GAPDH, 5′-CAAGGTCATCCATGACAACTTTG and 5′-GTCCACCACCCTGTTGCTGTAG.
(TIF)

Figure S3 **Mast cell degranulation profile in the heart of NTG and TG mice.** Mast cell were stained using toluidine blue method (A): a, b were shown with original magnification ×100; c, d were shown with original magnification ×400. Number of mast cell per section (B) and Number of mast cell per mm^2^ (C) were provided.
(TIF)
