# Supplementary figures and images for: Heparin-Binding EGF-Like Growth Factor Induces Heart Interstitial Fibrosis via an Akt/mTor/p70s6k Pathway
Source: PLoS One. 2012 Sep 12;7(9):e44946. doi: 10.1371/journal.pone.0044946 (PMC3440333; doi:10.1371/journal.pone.0044946)

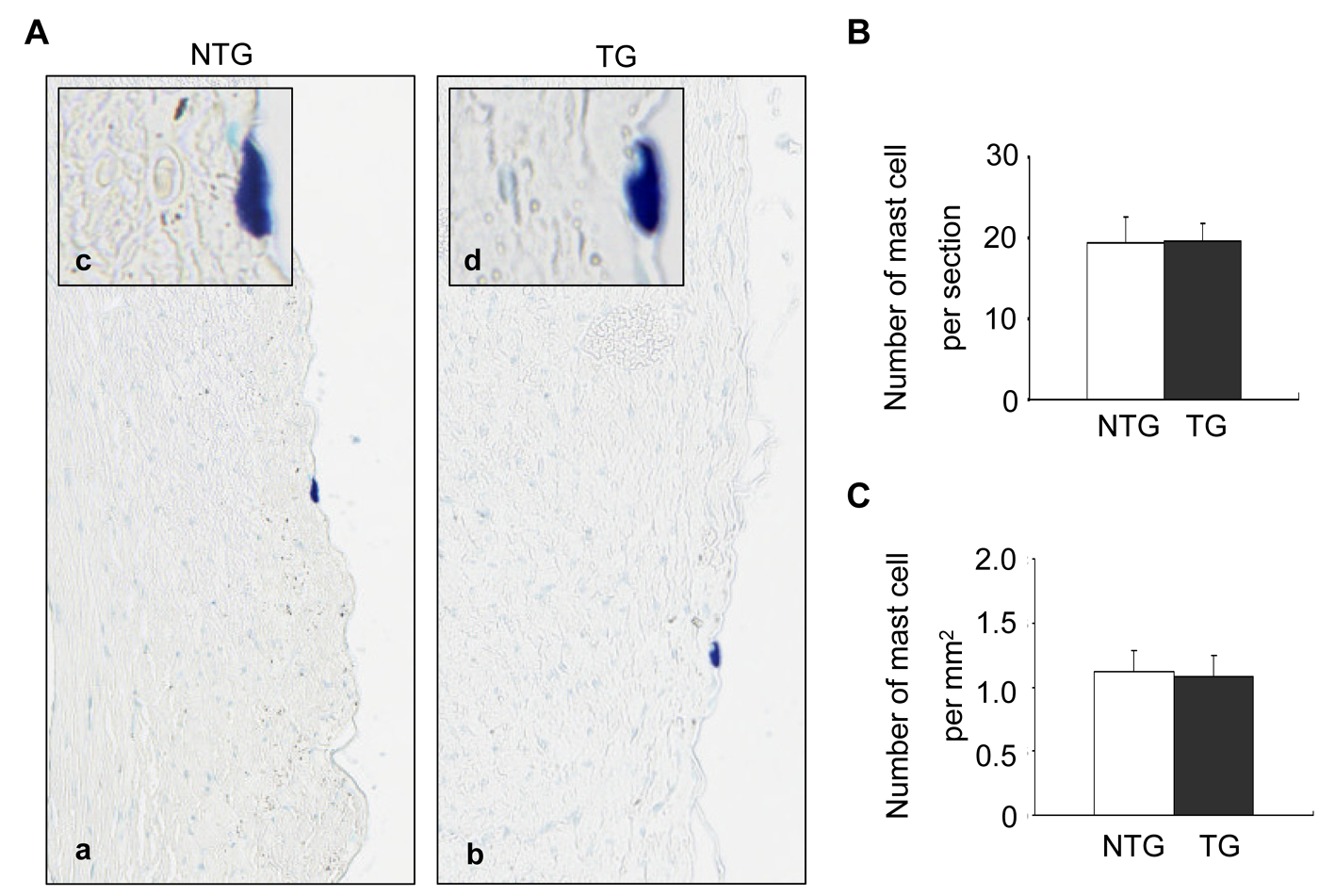

Supplement: Figure S1 — The phosphorylation levels of Akt (Ser308). Mice were sacrificed at 3 and 7 months of age, and heart-tissue samples were homogenized in RIPA buffer. Phosphorylation level of Akt (Ser308) was detected using western immunoblot. (TIF) [file pone.0044946.s001.tif]

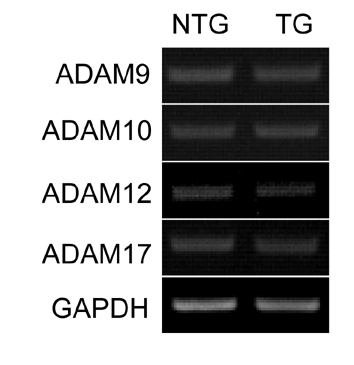

Supplement: Figure S2 — The mRNA expression levels of ADAMs in the heart of NTG and TG mice. Expression levels of mRNA for ADAM9, ADAM 10, ADAM 12 and ADAM 17were determined using RT-PCR, and GAPDH was used for normalization. Primers used included ADAM9, 5′- TGCCTCTCTGCGACTAAGGT and 5′- ACTCGGATGCTCCTCCTCAT; ADAM 10, 5′- AAACACCAGCGTGCCAAA and 5′- TTCAGCCAGAGTTGTGCGT; ADAM 12, 5′- GGGACCAGAGAGGAACTTACGA and 5′- CTTCTTGCCCGCATTTGA; ADAM 17, 5′- GTGGTTGGTGAGCCTGACTCTA and 5′- AAGCATCCTTCTCTTCGTTTGG; and GAPDH, 5′-CAAGGTCATCCATGACAACTTTG and 5′-GTCCACCACCCTGTTGCTGTAG. (TIF) [file pone.0044946.s002.tif]

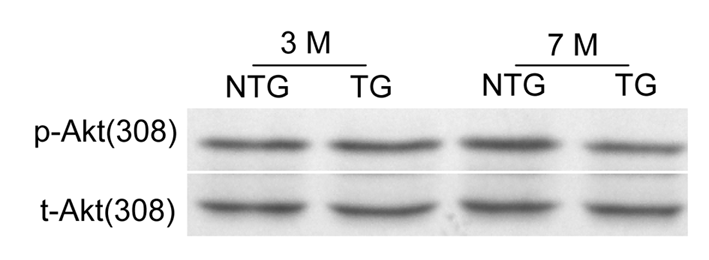

Supplement: Figure S3 — Mast cell degranulation profile in the heart of NTG and TG mice. Mast cell were stained using toluidine blue method (A): a, b were shown with original magnification ×100; c, d were shown with original magnification ×400. Number of mast cell per section (B) and Number of mast cell per mm2 (C) were provided. (TIF) [file pone.0044946.s003.tif]
